# Pro and Contra: Provocation Tests in Drug Hypersensitivity

**DOI:** 10.3390/ijms18071437

**Published:** 2017-07-04

**Authors:** Ozge Soyer, Umit Murat Sahiner, Bulent Enis Sekerel

**Affiliations:** Department of Pediatric Allergy Ankara, School of Medicine, Hacettepe University, Ankara 06100, Turkey; umsahner@yahoo.com (U.M.S.); b_sekerel@yahoo.com (B.E.S.)

**Keywords:** drug hypersensitivity reaction, provocation test, anaphylaxis

## Abstract

Drug provocation test (DPT) is the controlled administration of a drug to diagnose immune- or non-immune-mediated drug hypersensitivity and the last step for accurate recognition of drug hypersensitivity reactions when the previous diagnostic evaluations are negative or unavailable. A DPT is performed only if other conventional tests fail to yield conclusive results. In each clinical presentation, “to provoke or not to provoke” a patient should be decided after careful assessment of the risk–benefit ratio. Well-defined benefits of DPT include confirmative exclusion of diagnoses of drug hypersensitivity and provision of safe alternatives. However, disadvantages such as safety, difficulty in interpretations of results, lack of objective biomarkers, risks of resensitization, efficiency in daily practice, and lack of standardized protocols, are poorly debated. This review summarizes the current published research concerning DPT, with particular emphasis on the advantages and disadvantages of DPT in an evidence-based manner.

## 1. Introduction

Drug provocation test (DPT) is the controlled administration of a drug to diagnose immune- or non-immune-mediated drug hypersensitivity and the last step for accurate recognition of drug hypersensitivity reactions in the absence of informative alternative diagnostic tests (Table 1). DPT has the advantage of reproducing the allergic symptoms and any other adverse clinical manifestations regardless of the underlying mechanism. According to the European perspective, DPT is generally accepted as the “gold standard” investigation for the diagnosis of drug hypersensitivity [1]. However, from the American perspective, this approach is regarded as graded challenge (or test dosing), defined as the introduction of a medication cautiously so as not to induce a severe reaction [2].

Precision medicine is a developing field of medicine, based on disease endotypes, which are phenotypic subclasses associated with specific mechanisms underlying the disease. This approach requires the evaluation of a patient with a suspected drug hypersensitivity reaction from various perspectives including pharmacogenetics, diagnostics or therapeutic levels. Patients who are inappropriately classified as allergic to certain drugs can be shown to be tolerant in response to these drugs primarily via skin testing and drug provocations, revealing the precise diagnoses thereby reducing the expenses and side-effects of alternative therapies [3].

The provocative drug is either an alternative, a structurally/pharmacologically related drug or the culprit drug itself [4]. A DPT is performed if other less critical or less difficult tests fail to yield conclusive decisions. In each clinical presentation, “to provoke or not to provoke” a patient should be decided after balancing the risk–benefit ratio. Several factors may influence not only the decision but also the protocol for a DPT, such as the chronology of the index clinical reaction (immediate vs. non-immediate), the severity of the clinical reaction (anaphylaxis vs. mild reactions), the population involved (children vs. adults) and the facilities of the medical center (including intensive care unit) [5]. A definite diagnosis of drug hypersensitivity reaction, in fact, may become a clinical necessity that many drug courses may be required over a lifetime, usually as an emergency. The advantages and the disadvantages of DPT are summarized below (Table 2).

## 2. Pro: The Tip of the Iceberg

**Figure 1:**
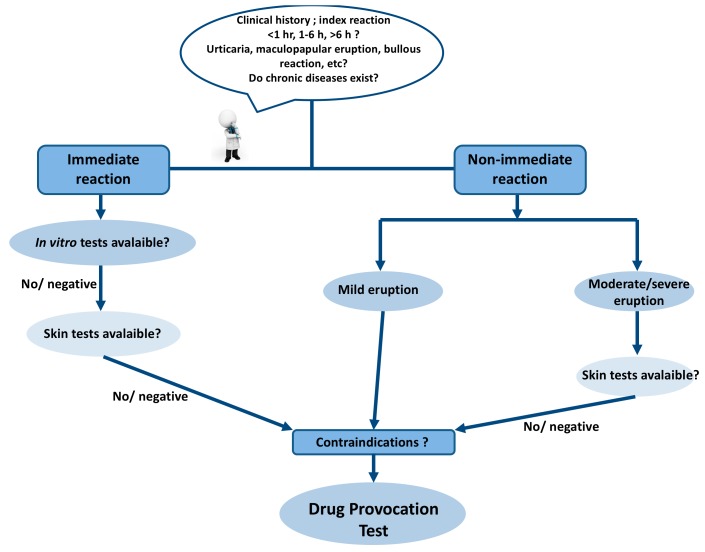
Diagnostic algorithm of drug hypersensitivity reactions.

An accurate history and description of clinical symptoms, as well as potential cofactors, provide hints for the underlying pathogenetic mechanisms and the selection of appropriate test procedures (Figure 1). However, there are many limitations during diagnostic work up, such as the obscure pathogenesis of certain reactions and the lack of optimal skin tests and validated in vitro tests for some drugs. In the case of a positive skin or an in vitro test, a relation between the drug and the hypersensitivity reaction can be established based on the clinical relevance. If the tests are negative or not available, it would be the time for application of DPTs. The clinician should decide on the aim of the DPT, whether it is to confirm or exclude the diagnosis of drug hypersensitivity or to find an alternative medication. The indications introduced by the European Academy of Allergy and Clinical Immunology (EAACI) will aid in decision making to plan and perform a DPT in different clinical situations [1].

### 2.1. Confirmation of Diagnosis

“To establish a firm diagnosis in suggestive history of drug hypersensitivity with negative, non-conclusive or non-available allergologic tests”

The history of drug hypersensitivity reactions should be evaluated on the basis of the underlying pathogenetic mechanisms. A definite history and characterization of the physical examination findings guide the selection of precise diagnostic test procedures. Drug provocation tests provide the valid evidence for the diagnosis of drug allergy, which is essential to determine the therapeutic future of the patients. In an initial study by Messaad et al., 1372 DPTs with a variety of drugs, including β-lactams, non-steroidal anti-inflammatory drugs (NSAID), macrolides and quinolones, were performed in 898 patients with suspected immediate drug allergy. Drug allergy was confirmed in 17.6% of patients who demonstrated similar symptoms during DPT, although milder and of shorter duration [6]. Dona et al. confirmed the drug allergy in 1683 of 4460 patients by clinical history (44%), skin tests (14.6%), in vitro testing (10.4%), and by DPT (30.8%). Thus, DPT was required to establish a diagnosis in one out of every three patients [7]. Overall, patients with drug hypersensitivity reactions (DHR) worth testing with a standardized workup including DPTs, which result in definitive advice concerning future tolerability, avoidance of certain drugs, and recommendations for alternative medications in majority of patients [8]. Drug hypersensitivity reactions also had social impact on patients’ life. They were fearful of recurrence of the previous reactions and/or permanent impairment of the body, their health, and thereby limitation of their social activities. Precise diagnosis of DHR would simply decrease the social and medical burden of drug allergy [9].

Initial skin testing with the culprit drug prior to the provocation test is considered a safe, reliable, and practical approach for the assessment of suspected DHRs. Well-defined nonirritating concentrations are currently in use during skin prick tests and intradermal tests (IDTs) for specific drugs, such as β-lactam antibiotics, chemotherapeutic drugs, and some of the biological agents. However, there is still a lack of information regarding the standardized concentrations of non-β-lactam antibiotics including macrolides used in skin tests [10]. In a pediatric study, using the IDT concentrations of clarithromycin which were verified in an adult population [11], nine out of twenty children showed false positive IDT who had negative DPTs. However, two patients with negative skin tests developed urticaria during DPTs. Younger age might contribute to false-positive IDT results, and the irritating effect of drugs appears to be more pronounced in children, which would in turn increase the requirement for performing DPTs [12]. Most of the hypersensitivity reactions with iodinated contrast media are associated with the histamine release from basophils and mast cells and the IgE-mediated allergic mechanism can be demonstrated only in a minority of cases [13,14]. Provocation tests with progressive increase of the injected dose of the radiocontrast media over several days are effective to exclude a drug hypersensitivity diagnosis [15,16]. Recently, it was shown that, when the results of skin tests and intravenous provocation with low-dose radiocontrast media were negative, the risk and severity of reaction was low in the case of contrast media re-exposure [17].

Drug provocation test results are particularly crucial for diagnosis of NSAID hypersensitivity because of the insufficiency of diagnostic skin and in vitro tests (except for IgE-mediated reaction to dipyrone), the significant potential to induce anaphylaxis and the increased prevalence of hypersensitivity reactions in certain risk groups, such as chronic urticaria [18,19,20,21,22]. NSAID hypersensitivity was shown in 7.6–68.2% of children and 10.7–83.2% of adults as they had positive DPT results to the culprit NSAID [23,24,25,26,27,28,29]. In addition, DPTs are also important in the diagnosis of hypersensitivity reactions of other drugs including proton pump inhibitors, fluoroquinolones, and local anesthetics [30,31,32]. The implementation of DPT with antineoplastic and biological agents not only aid in the diagnosis of DHR, but also prevents unnecessary desensitizations in non-hypersensitive patients (30–56% of patients, depending on the culprit-drug) [33].

In children with a history of β-lactam hypersensitivity reaction, the evaluation of drug allergy led to the confirmation of drug hypersensitivity in 6.8–15.9% of patients [34,35,36,37]. In children with chronic diseases such as cystic fibrosis, this ratio increased to 47.3% [38]. Skin test positivity is 22.2–79.1% in patients with immediate β-lactam hypersensitivity [36,39]. The specificity of the measurement of β-lactam specific IgE was high (71.4–97%), but the sensitivity (25–42%) was low [40,41], whereas the sensitivity increased (75%) in a group of patients with a clinical history of anaphylactic shock and negative skin test results. In non-immediate reactions such as maculopapular eruptions, the prevalence of true β-lactam hypersensitivity declines particularly in children [42]. The diagnostic yield of tests including patch tests and delayed readings of IDT are low [43]. Caubet et al. assessed 88 children with a history of late onset urticarial and maculopapular rashes due to β-lactam use, eleven (13%) of whom demonstrated positive ID testing. Six children (6.8%) had positive oral DPT with β-lactams presenting as mild cutaneous rashes, mostly as late reactions (7–12 h later). Four of the six children who had positive DPT also showed positive ID test results with β-lactams. They concluded that DPTs should be considered in all children, who develop a delayed-onset benign rash as urticarial or maculopapular during treatment with a β-lactam antibiotic [34]. In the absence of antecedent skin testing, Vezir et al. implemented direct oral provocation with culprit antibiotics for five days to patients with non-immediate mild cutaneous reactions without systemic symptoms caused by β-lactam antibiotics [42]. Four patients (3.4%) developed urticaria, revealing positive DPT results beyond any severe reactions [42]. The age of the patient also contributes to the decision of a diagnostic pathway, such as the preference of direct drug provocation in younger children, where skin testing could be painful and difficult to perform [44]. Recently, the Pediatric Task Force of the EAACI Drug Allergy Interest Group suggested the use of DPT without prior skin testing in the cases of non-immediate mild cutaneous reactions [45]. This approach may also reduce the cost of drug allergy algorithm, up to 35% per patient [46].

The misclassification of particularly hospitalized patients as “drug hypersensitive” leads to the unnecessary use of alternative medications that are often more expensive and less effective. In a group of hospitalized patients, the suspicion of drug allergy resulted in the substitution of β-lactam antibiotics with quinolones, or vancomycin and NSAIDs with coxibs, or that paracetamol and iodinated contrast media were not used despite indications. These changes in the use of drugs led to a fourfold increase in the mean of the costs of treatment. However, the allergologic work up revealed that in 32% patients, the original diagnosis of drug hypersensitivity was correct [47]. The ambiguous penicillin allergy has higher burden on patients’ health in terms of the microbial resistance patterns, and the treatment costs. Solensky et al. reported that physicians usually preferred cephalosporins, macrolides, quinolones, tetracyclines, nitrofurantoin to manage the patients having penicillin allergy documented in their medical records. The type of the penicillin allergy history and the severity of the disease process influenced the choice of antibiotics [48]. Alternative antibiotics usually have broader spectrum, have potential for inducing antibiotic resistance, are more expensive and may be associated with higher rate of clinical failure [49,50,51,52,53].

### 2.2. Exclusion of Cross-Reactivity

“To exclude cross-reactivity of related drugs in proven hypersensitivity”

Drug provocation tests are essential to characterize cross-reactivity in drug hypersensitive patients. A search for well tolerated alternative β-lactam is advised in cases of diagnosed β-lactam allergy [54]. The in vitro tests, such as basophil activation test, are not sufficient to differentiate selective reactors and cross-reactors in patients with immediate allergic reactions to β-lactams [55]. Blanca-Lopez et al. defined a group of patients who were selective responders to amoxicillin but could tolerate penicillin G and penicillin V [56]. In an adult population with a proven β-lactam allergy, 11.9% of patients were sensitized to both penicillins and cephalosporins. The prevalence of cefuroxime allergy was 6.3% (4.2% diagnosed by DPT) in patients sensitized to β-lactams [57]. Zambanino et al. demonstrated by using DPT in a group of children with immediate or non-immediate hypersensitivity to amoxicillin or amoxicillin clavulanic acid, that cefuroxime was tolerated by all patients [36]. Cross-reactivity seems higher in immediate reactions when penicillins and cephalosporins are identical or similar in the R1 side chain [58]. However, in a comprehensive study including 102 patients (89 of subjects had an anaphylactic reaction with a cephalosporin in their medical history) with at least one cephalosporin hypersensitivity confirmed by positive skin test responses, it was shown that cephalosporin hypersensitivity was unlikely to be a class hypersensitivity. They performed 326 DPTs with alternative cephalosporins in the case of negative skin test results, which were all tolerated. The two subclasses of cephalosporins were identified: those with a methoxyimino group in their R1 side chains plus ceftazidime, and aminocephalosporins. Nevertheless, pretreatment skin tests with alternative cephalosporins and DPTs were needed [59]. The observed cross-reactivity between penicillins, carbapenems, and/or aztreonam was low therefore, skin tests and DPTs showed negative results in majority of the cases to exclude cross-reactivity [60,61,62,63].

Drug provocation tests are specifically important to classify patients with NSAID hypersensitivity. NSAID hypersensitive subjects mainly exhibit cross reactivity to other NSAIDs, thus are designated as cross intolerant whereas, single NSAID hypersensitivity is termed as selective responder [64,65]. In a multicenter study where DPTs were applied to various NSAID hypersensitivity cases, a mean of 1.71 ± 0.73 oral challenges were performed in the cross intolerant group and 2.93 ± 1.11 in the selective responder group for diagnosis. Cutaneous symptoms were observed more frequently by the cross intolerants, whereas anaphylaxis by the selective responders [64]. When the patients demonstrated typical presentations such as asthmatic reaction, multiple reactions to multiple NSAIDs, or anaphylaxis to a single NSAID, DPT was needed to confirm the tolerability to alternative compounds. Corzo et al. reported that all children with positive DPT results with aspirin and the culprit NSAID tolerated paracetamol and etoricoxib (a selective cyclooxygenase 2 inhibitor), 4.9% developed a reaction to meloxicam (preferential cyclooxygenase 2 inhibitor) during DPT [66]. Among 30 children with NSAID hypersensitivity, two children reacted to aspirin, ibuprofen, and paracetamol, as well as one to nimesulid during DPT [23]. Celik et al. demonstrated in 309 adults with NSAID hypersensitivity that cyclooxygenase 2 inhibitors were well tolerated in the majority of the patients (nimesulide: 91.9%; meloxicam: 90.2%; rofecoxib: 94.9%; and celecoxib: 94.9%). Twenty-five patients reacted to cyclooxygenase 2 inhibitors during DPT, of which eight cases developed bronchospasm and one case exhibited anaphylaxis [67]. Meloxicam and etoricoxib were found to be safe alternatives for the aspirin-hypersensitive patients with asthma and/or nasal polyps [68,69].

Time-saving and cost-effective approaches to find safe alternatives during diagnostic work up of DHR were proposed. Kalyoncu et al. tested three alternative drugs (meloxicam, rofecoxib, paracetamol) a day in NSAID hypersensitive patients and the time required to perform these tests decreased by 56% [70]. Likewise, triple tests performed with antibiotic and NSAID on the same day for multidrug hypersensitive patients with non-life-threatening allergic reactions seems to be safe and time-saving [71].

### 2.3. Safe Alternative

“To provide pharmacologically and/or structurally non-related safe drugs in proven hypersensitivity”

In the case of β-lactam hypersensitivity, a physician might choose alternatives like macrolides or quinolones as structurally distinct from the agent that had caused the reaction during the course of management of patients. Due to the limited data on the sensitivity and the specificity of skin tests and in vitro tests of non-β lactam antibiotics, DPTs are usually performed to prove tolerance [12,31,72,73]. Multiple drug allergy syndrome (MDAS) is defined as adverse reactions to two or more structurally unrelated drugs with an underlying immune-mediated mechanism causing the reaction [74]. MDAS is further divided into two subtypes: the development of hypersensitivity reactions with different drugs given simultaneously and the progression of drug sensitizations in a sequential manner [75]. Although some of these patients with MDAS exhibited severe cutaneous drug adverse drug reactions, a drug allergy work up including DPT might be needed not only to confirm the diagnosis but also to find unrelated alternatives as indicated under close supervision [76,77]. Albeit meticulous, this approach might relieve the anxiety of subjects who would refuse to take the indicated drug without a proof of tolerance.

### 2.4. Exclusion of Diagnosis

“To exclude hypersensitivity in non-suggestive history of drug hypersensitivity and in patients with non-specific symptoms”

The most common indication of DPT reported in the survey of World Allergy Organization concerning clinical practices in drug hypersensitivity was to exclude hypersensitivity where the history was not suggestive [78]. The majority of patients are over diagnosed as drug hypersensitive based on a suggestive history without proof by appropriate tests. In a community-based drug hypersensitivity prevalence study in children, the rate of the parent-reported immediate type drug hypersensitivity was 7.87%. The clinical history suggestive of drug allergy was for 1.16% of the cases after a phone survey, whereas the true frequency of immediate type drug hypersensitivity was demonstrated to be 0.11% [79]. Drugs such as local anesthetics may cause psychomotor symptoms frequently concerning vasovagal response, hyperventilation and panic attack and anxiety which can be misdiagnosed as a hypersensitivity reaction [80]. The IgE-mediated reactions to local anesthetics are unusual nevertheless many patients are identified as hypersensitive to local anesthetics [81,82]. In order to exclude the diagnosis of hypersensitivity to local anesthetics, skin testing followed by graded challenge tests can be performed with the same local anesthetic that is intended to be used [83].

### 2.5. Natural Course

Drug provocation tests may be required in the assessment of natural course of drug hypersensitivity reactions. In studies concerning patients with immediate hypersensitivity to penicillins or cephalosporins, skin tests are more likely to show negative results due to the time elapsed between the clinical reaction and the application of the skin test [84,85]. In the case of in vitro methods, drug specific IgE measurements show negative results later than drug specific basophil activation tests [86]. However, in these studies DPTs were not performed to evaluate the development of clinical tolerance. Recently, Dona et al. reported that 63% of patients who exhibited NSAID induced urticaria and angioedema established tolerance 72 months after the last reaction, confirmed by NSAID DPT [87]. There is no single study that followed the natural course of a DPT-proven antibiotic allergy with further investigations [88].

## 3. Contra: Bottom of the Iceberg

It cannot be disputed that DPTs represent a potential risk to the patient, are time consuming and need appropriate medical facilities (including access to an intensive care unit) and trained personnel [1]. The person undergoing the DPT should be healthy on the day of testing, with no sign of allergy, infection, exacerbation of a disease that can stimulate an immune response, preferably not use of any other medication. These conditions might be difficult to attain in chronically ill patients. A negative DPT result does not confirm tolerance to the drug in the future, instead indicates that there is no drug hypersensitivity reaction at the time of the provocation and to the maximum doses used during the provocation [89].

The classification of DHRs is challenging because for many drugs and clinical manifestations of these reactions, the underlying mechanisms are poorly understood. Clinically, DHRs are classified as “immediate” with typical symptoms of IgE mediated reactions such as urticaria, angioedema, rhinitis, bronchospasm, anaphylaxis, etc. which occur within 1–6 h after the last drug administration, and as “non-immediate” with clinical involvement of the skin (i.e., delayed urticaria, maculopapular eruptions, vasculitis, bullous eruptions, etc.) and/or internal organs (hepatitis, renal failure, anemia, neutropenia, etc.), which occur 1 h to some days after the initial drug administration [90]. The choice among the skin tests, in vitro tests and DPTs to achieve a definitive diagnosis is mainly based on the clinical classification of the index reaction. The DHR history should include the symptomatology, the route of the administration of the drug in consideration, the timing of the symptoms (previous exposure, period between the last dose and the onset of symptoms and days of the treatment, effect of the cessation of drug, medications to treat DHR and the response to them), and other medications taken (both at the time of the reaction and other drugs of the same class used ever since), [90]. Physicians are obliged to rely on the clinical history recalled by their patients, or parents in the case of children. It is also difficult for patients to remember all the important details/issues. The physician sometimes may not differentiate an immediate from a non-immediate reaction if the subject cannot remind the chronology of the reaction with mild presentation or only urticaria; likewise the physician cannot discriminate between a maculopapular eruption and cutaneous vasculitis from the recalled history (Box 1). Hjortlund et al. demonstrated that a significant number of patients could not remember the time interval between the drug intake and reaction (15.7%) or even the name of culprit drug (18.4%) [91].

Box 1 **Patient with a history of pink eruption.**

A seven-year-old girl was admitted to our pediatric allergy outpatient department with a history of DHR. One year ago, she developed a pink, itchy rash on trunk and extremities on the ninth day of amoxicillin-clavulanic acid treatment. The therapy was discontinued, however, the family could not remember how long the resolution period was and the medications used. In our department, prick and intradermal skin tests were performed on the forearm skin, with penicilloyl polylysine (PPL) and the minor determinant mixture (MDM) (Diater, Madrid, Spain), as well as amoxicillin-clavulanic acid (20 mg/mL). Both immediate and delayed readings (48 and 72 h) of intradermal tests were negative. The patch test with amoxicillin-clavulanic acid (10% petrolatum, occlusion time 48 h) also revealed a negative result. We performed an extended oral DPT with amoxicillin-clavulanic acid and on the fifth day, the parents noticed the onset of an eruption on her legs. The physical examination revealed multiple purpuric plaques on the posterior of both legs compatible with cutaneous vasculitis. All laboratory tests (complete blood cell count, C reactive protein, sedimentation rate, renal and liver function panel, urine analysis, antinuclear antibody, ANCA, complement levels (C3 and C4)) were unremarkable. The parents did not consent for a skin biopsy. The drug was withdrawn, methylprednisolone (2 mg/kg/day) was administered for three days and the skin lesions resolved within 10 days (The subjects gave informed consent for inclusion. The study was conducted in accordance with the Declaration of Helsinki, and the protocol was approved by the Ethical Committee of Hacettepe University; HEK 12/50-12 (11 May 2012))

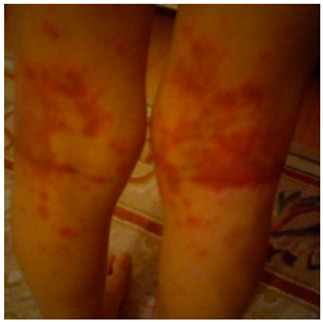

Studies featuring the negative predictive value (NPV) of DPTs are encouraging. In a multicenter study concerning NPV of DPTs with β-lactams, 457 patients with either immediate or non-immediate reactions to β-lactams were accessed at least six months after a negative drug allergy workup, including DPTs. One hundred eighteen (25.8%) of a total of 457 patients underwent a DPT with the previously suspected β-lactam or one of the same class. Only nine patients (7.6%) experienced a mild reaction after more than 1 h of drug intake. In addition, five urticaria, three exanthema and one undefined cutaneous reaction cases were observed. Only four of the patients consented to a consecutive DPT. However, two of these four patients displayed a rash upon the second exposition, resulting in a NPV of DPTs with β-lactams of 94.1%. Two of the patients displayed negative results in allergy re-evaluations, so that the NPV of DPTs with β-lactams was 94.1%. None of the patients showing false negative results experienced a life-threatening reaction [92]. In children, Capanoglu et al. performed DPTs four weeks after the first evaluation in 71 patients who had negative results of initial DPTs with β-lactams (71.8%, non-immediate reaction) and only 2 patients (2.8%) reacted with maculopapular eruptions [93]. In another study of the same group, NPV of DPTs with diverse drugs was found to be 95.6% [94]. The NPV of DPT with NSAIDs was higher (97.8%) [95].

The resensitizations, the presence of cofactors, drug interactions and false-negative test results are among the possible explanations for a positive reaction after a DPT with negative results. Hershkovich et al. repeated skin tests and DPTs 1–5 months after the first negative diagnostic work up for β-lactam hypersensitivity. Two patients (2%) had positive intradermal test results with penicillin, and one patient developed a maculopapular rash by DPT [96]. A similar outcome was observed by Mendelson et al. who re-evaluated 219 children with prior negative workup results 4 weeks later, and the frequency of positive skin test results was 0.9% [97]. Children and adolescents with suspected β-lactam hypersensitivity were evaluated with a two-step protocol; 247 subjects underwent skin tests with culprit drugs. Fifty-three (21.5%) of the 247 skin tested children had an initial positive reaction. Extended DPTs for 10 days were performed on the remaining 194 patients, five of whom revealed positive results. After completion of the oral challenge, 189 children returned for a second skin testing; 26 of which turned to show positive results, leading to a resensitization rate of 13.7%, which was 10.5% for the overall study population [98].

There are many limitations which prevent DPT to be a part of the routine clinical practice. Although DPT is considered as the “gold standard” for the diagnosis of drug hypersensitivity reactions, such tests are interfered by the risk of life-threatening reactions and contraindicated in severe drug reactions (i.e., bullous drug eruptions, systemic vasculitis, blood cytopenia, nephritis, etc.) [1] in patients using β-blockers or ACE-inhibitors or, are troublesome for patients with hypersensitivity to neuromuscular blocking agents [99] (Table 3). In the case of perioperative anaphylaxis, DPT with general anesthetics cannot normally be performed because of the pharmacological effects of these drugs [100].

The accurate diagnosis of the drug hypersensitivity reactions is essentially important in patients with chronic diseases or other clinical conditions, which require the use of medications for survival or the improvement of the quality of life. Nevertheless, these circumstances (such as heart and lung diseases, infections, psychiatric disorders) might represent the contraindications in one out of ten patients for diagnostic work up including DPT [7]. DPT is not indicated when the culprit drug is unlikely to be needed and structurally unrelated alternatives exist or in the case of a severe concurrent disease or pregnancy [101].

### 3.1. Safety Issues

The severity of a future reaction after drug hypersensitivity reactions cannot be precisely anticipated so a careful evaluation of the requirement for a DPT and the determination of the dosage of the drug is essential. A DPT due to a history of drug induced urticaria may end up with severe anaphylaxis (Box 2).

Box 2 **Patient with a history of urticaria exacerbation.**

A ten-year-old boy was admitted to our pediatric allergy outpatient department with a history of chronic urticaria. Six months ago, within two hours of ibuprofen use due to high fever, he had an exacerbation of urticaria without any symptom of other system involvement. His condition improved upon the administration of antihistamines and systemic corticosteroids in the emergency department. Because of the concomitant disease of chronic urticaria, and that infections might exacerbate urticarial rashes, we planned an aspirin provocation test with doses of 10, 17, 44 and 117 mg every 1.5 h [102]. Within ten minutes of taking 44 mg aspirin, the patient developed an immediate generalized urticaria and angioedema. Since the reaction progressed so quickly, adrenaline (0.01 mg/kg/dose, im) was administered. Afterwards, the patient presented with severe anaphylaxis with a chronology of stridor, wheezing and diarrhea and was treated with high flow oxygen, nebulized adrenaline, and a second dose of intramuscular adrenaline, methyl-prednisolone and parenteral antihistamines. The patient was transferred to the intensive care unit. His stridor recovered after 4 h.

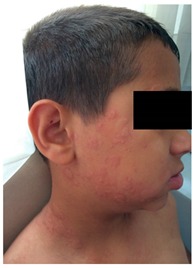

Many studies concerning drug provocation tests did not report the clinical presentations of positive results in detail [7,25,64,103,104,105,106]. Some of them declare the findings did not underline the diagnosis of anaphylaxis. In a study dealing with cross reactivity of penicillin derivatives, 11 patients (20%) had positive DPT with amoxicillin or amoxicillin-clavulanic acid and eight of them exhibited at least two system involvements such as generalized cutaneous pruritus, wheals and throat tightness or cough or hypotension [56].

The prevalence of anaphylaxis during a DPT may differ between studies. According to the largest study of DPT, including 1913 episodes and prolonged provocations with culprit drugs, only one patient demonstrated severe circulatory and respiratory symptoms fulfilling the criteria for anaphylaxis requiring adrenaline [107]. The rate of anaphylaxis that exhibited in the course of NSAID provocations was 3.2–12.3% in childhood [24,108,109]. Zambonino et al. reported nine cases of anaphylaxis treated with adrenaline in 73 positive NSAID provocation tests [24]. As a result of 39 positive NSAID provocation tests, we experienced two cases of anaphylaxis that required the administration of adrenaline and both patients responded quickly to the injection. Spirometry was used periodically during OPTs to monitor patients. The fall in FEV1 was a warning sign (even in the absence of respiratory symptoms) to stop the DPT to avoid severe and long-lasting reactions in four additional patients, who otherwise would receive extra doses to complete the provocation test. A close monitoring with spirometry is a complementing method to prevent severe hypersensitivity reactions when performing DPTs with NSAIDs [23]. In a previous study, the involvement of the respiratory system was reported with a higher frequency. Among the clinical presentation of 19 positive DPT with NSAID periorbital angioedema (100%), wheezing (38%), urticaria (38%) and rhinorrhea (23%) were observed in children. The respiratory symptoms of tachypnea, wheezing, and coughing developed in three of eight patients with prior diagnoses of asthma [110].

The severity of the positive reaction during DPT seems to be affected by the type of drug used for provocation. In a 20-year period, among the 1431 children assessed as showing β-lactam hypersensitivity, 130 (48.9%) of 227 DPTs revealed positive results. However, in 88 reactions that were reported, challenge tests were performed at home. All reactions were mild to moderately severe; responded well to medication, except for one urticaria with asthma exacerbation and two severe serum sickness-like reactions [35]. Anaphylaxis is rarely reported upon DPTs in patients with β-lactam hypersensitivity [57,111,112,113]. Skin tests as a prior step contribute to the increased safety of diagnostic work up of DHR. However, recently, in a large cohort of positive DPT with β-lactams (*n* = 182), anaphylaxis with or without shock were observed in 4 (2.2%) and 21 cases (11.5%), respectively [5].

In some studies, no anaphylaxis or severe hypersensitivity reaction were observed upon DPT [36,66,114,115,116,117]. There is limited number of studies with the aim to evaluate the safety of DPT. In a study concerning a diverse group of the drug hypersensitivity reactions (HSR), two out of 243 DPTs caused severe reactions: cephalexin-induced anaphylactic shock and bupivacaine-induced anaphylaxis without shock [118]. Iammatteo et al. compared the safety and outcomes of one- and two-step test doses with multistep provocations among patients with low risk history of the drug HSR [119]. The test dose protocol was implied such that patients would receive one-tenth of the full dose for a parenteral medication or one-fourth of a pill for an oral medication followed by the full dose after 60 min of observation. The adverse drug reaction rates were similar; 11% for test doses and 12% for multistep provocations. The conclusion was that one- and two-step test doses were safe and multistep provocations did not provide additional safety in appropriately selected patients [119].

### 3.2. Standardization of DPT

There is usually a lack of uniformity not only in selection of the diagnostic tests but also in management of drug allergy in daily practice of clinicians around the world. In a survey study, distributed through the International Allergy Societies, it was stated that 64% of respondents considered DPT extremely useful for both the exclusion and the confirmation of β-lactam allergy. Nevertheless, the methodology of how to conduct challenges, for example, the dosing of the antibiotic during a challenge on the first day, how to choose alternative drug in the case of amoxicillin allergy, and the location of DPT were not consistent [120].

Regardless of the increasing number of studies supporting the role of DPTs as a diagnostic method, very few emphasize the necessity of the standardization of the protocols; rather consensus reports recommend the algorithms [1,2,65,121]. This leads to a variety of protocols from different centers and sometimes with different test results [6,35,91,106,122]. One of the main discrepancies involves the duration of provocation tests in non-immediate drug hypersensitivity reactions, particularly related to β-lactams as to whether one-day DPT protocol is sensitive enough (vs. several-day DPT). Chiriac and colleagues tested the safety of a one-day protocol for immediate and mild non-immediate reactors of β-lactams, for both children and adults and followed the subjects for 48 h. Ninety-one cases (50%) had reacted during Day 1 while 57% of these cases were immediate reactors that reacted within one hour of drug administration. When reactive time points were set as 24 h, 48 h, and 72 h, the percentage of the match of index reaction with the chronology of the DPT was over 95% for all case groups. However, there are also many reports contradicting this approach. Borch et al. performed 10-day penicillin provocations for histories of non-immediate reactions and found that 50% experienced a cutaneous reaction on Day 6 on average [123]. In another study, seven-day protocol improved the diagnosis in the 11.7% (13 of 111) of those having a positive outcome on penicillin challenge [91], while 2.3% were improved by a five-day protocol [124]. A large population of patients that were subjected to prolonged DPTs showed 11% positive test results and only one of five patients tested positive on the first dose [108]. In addition, more patients who underwent the extended DPT (78%) used the drug after diagnostic workup again compared to patients with a short DPT (61%) [125].

### 3.3. Interpretation of DPT

Drug provocation test results should be assessed based on objective parameters; nevertheless, subjective symptoms should also be recorded. Clinical presentation, as well as the progress of a reaction over time, should be documented and, where possible, quantitative parameters should also be measured (e.g., blood pressure, respiratory parameters, and serum tryptase levels) [126]. Patients who exhibited previous pseudoallergic reactions and underwent double blind placebo controlled DPTs developed similar symptoms, including increase blood pressure, tachycardia, shortness of breath, restlessness mainly due to increased anxiety during the provocations of drug and placebo [127].

The nocebo effect is characterized as a negative and troublesome response to the placebo, which is an inactive substance or a procedure. Nocebo responses might be classified as subjective (e.g., sensation of dyspnea, nausea, headache, and itching) and objective (vomiting, tachycardia, changes in blood pressure, skin rashes, and wheezing) [128]. A placebo controlled DPT in 600 adult patients revealed that the administration of placebo provoked untoward reactions in 27% of patients. The majority of these reactions were subjective symptoms regarded as disturbing by all subjects [129]. Bavbek et al. reported that non-atopy, high education level and older drug hypersensitivity reaction histories were associated with nocebo effect during DPTs [128]. In open DPTs, nocebo-like responses might lead to the misinterpretation of mainly subjective symptoms as a positive DPT result. These findings underline the necessity to perform a placebo controlled DPT, especially in adult subjects, particularly in females and in patients with multiple drug allergies [130].

The difficulty of the assessment of adverse reactions during DPT creates the requirement of biomarkers to make a firm decision. Sala-Cunill et al. demonstrated that serum tryptase levels were more frequently elevated in severe anaphylaxis, with a positive correlation between the grades of severity and the levels of tryptase. In patients with drug induced anaphylaxis, the serum tryptase levels were higher than in patients with food induced anaphylaxis. However, in the 36.3% of patients with clinically defined anaphylaxis, the tryptase levels remained low during an acute episode [131]. The investigation of anaphylaxis during general anesthesia is challenging. The measurement of serial serum tryptase is a valuable tool in the evaluation of anaphylaxis in operating rooms and >33.6 μg/L or a five-fold increase from the baseline derives a positive predictive value (PPV) ≥85% [132]. These findings implied tryptase as a potential biomarker for monitoring of DPTs. However, Komericki et al. showed in 34 patients with positive DPT results that the serum tryptase increased in seven (20.5%), remained similar in 11 (32.3%), and decreased in 16 (47%) of the patients. Thus, serum tryptase was not useful to differentiate mild allergic reactions from the conversion symptoms [133]. In addition, serum basal tryptase is not a risk factor for immediate-type drug hypersensitivity during childhood [134].

### 3.4. Efficiency of DPT

Patients with drug hypersensitivities exhibited a certain level of anxiety and depression attributed to their past experience of reactions after consumption of drugs [135]. In addition, most patients who underwent DPTs have a significant amount of anxiety before the tests, which gradually decreased after receiving negative test results [136]. Gomes et al. reported that most patients accepted DPTs for diagnostic purpose regardless of the test results, and 95% of them believed that DPTs were useful and they would recommend DPT to others [137]. However, the exclusion of NSAID hypersensitivity diagnosis by DPT was not sufficient to convince the patients that the majority of patients with a history of urticaria/angioedema avoided the intake of the tested NSAIDs due to the fear of possible new reactions [138]. In a multicenter study including patients who exhibited DHR with NSAID, antibiotics or neuromuscular blocking agents, 22.8% of the participants avoided not only the use of the culprit drug but also other drugs [9]. General practitioners might also refuse repetitive prescription of the drug even after exclusion of hypersensitivity [139].

**Figure 2:**
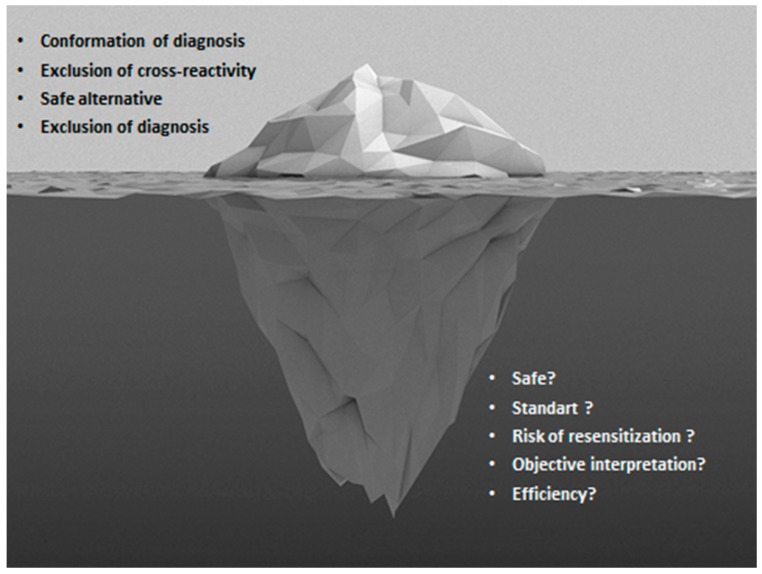
The tip and the bottom of the iceberg in drug provocation tests.

In summary, drug allergy is increasing in the 21st century and we are encountered with numerous challenges during the management of patients with drug hypersensitivity reactions [140]. DPT is still required for the accurate diagnosis of many drug HSRs, as well as to evaluate the tolerability of alternative medications. The DPT will preserve its valuable contribution in the drug allergy management until better alternatives are proposed due to the risks it poses, costs, the time required, and the need for experienced healthcare personnel and service (Figure 2). There is still a gap in the standardization of procedures of DPT and interpretation of test results. Despite negative DPT results, implementation of the culprit drug in routine clinical practice is sometimes lacking. As experience grows, the development of shorter, inexpensive and less risky DPT methods will lead to a better quality of the health service and thus the established application of these methods.

## Figures and Tables

**Table 1:** Drug provocation tests.

Definition: Controlled administration of a drug to diagnose drug hypersensitivity and the last step for accurate recognition of drug hypersensitivity reactions if the previous diagnostic evaluations are negative or unavailable.
Requirements: • Trained personnel, who know how to perform tests, are ready to recognize and treat symptoms and signs of a hypersensitivity reaction • Equipment for emergency resuscitations
Methods • Informed consent • Commercial agents are usually used • Route of administration: Oral, parenteral, cutaneous, bronchial, etc. • Starting dose depends on severity and immediate/non-immediate timing of index reaction (1/10000–1/10) • Interval: 30–90 min

**Table 2:** Advantages and disadvantages of Drug provocation tests.

Advantages	Disadvantages
Confirmation or exclusion of diagnosis of drug hypersensitivity	Potentially dangerous
Less use of more expensive alternatives	DPT protocol is chosen based on patients′/parents′ report about the reaction suffered
Less use of broad spectrum antibiotics, decreased risk of antibiotic resistance	False positive and false negative results may occur
Reduced cost of drug allergy algorithm	Cofactors may be absent
Generally good safety profile	Potential risk of resensitization
Acceptable for most patients	Although gold standard, many contraindications to perform DPT may be present
Avoidance of unnecessary desensitizations	Lack of standardized protocols, especially for non-immediate reactions
Provision of safe alternative	Subjective symptoms could be difficult to interpret
Decreased social burden of drug allergy	Lack of objective and reliable biomarkers (e.g., serum tryptase)
	Negative results may not be sufficient to reuse the culprit drug
	Need experienced personnel and well-established clinical settings

**Table 3:** Circumstances in which DPTs are contraindicated or not preferred.

Patient related factors • Uncontrolled asthma • Uncontrolled underlying chronic disease • Pregnancy • Use of β-blockers • If underlying heart disease is a contraindication for the use of adrenaline
Index drug hypersensitivity reaction • Vasculitis syndromes • Bullous exanthemas (Steven Johnson syndrome, toxic epidermal necrolysis, bullous fixed drug eruptions, etc.) • Acute generalized exanthematous pustulosis • Drug-induced autoimmune disease (systemic lupus erythematosus, pemphigus vulgaris, etc.) • Drug induced hypersensitivity syndromes (DRESS) • Involvement of specific organ systems (hepatitis, nephritis, blood dyscrasias, etc.) • Severe anaphylaxis
Culprit drug • Unlikely to be needed and several structurally unrelated alternatives exist
